# A Rare Case of Splenic *Pneumocystis jirovecii* in a HIV-Positive Patient

**DOI:** 10.1155/2020/8509591

**Published:** 2020-06-10

**Authors:** Hafsa Abbas, Harish Patel, Ahmed Baiomi, Masooma Niazi, Trupti Vakde, Sridhar Chilimuri

**Affiliations:** ^1^Division of Gastroenterology, BronxCare Hospital Center, Bronx, NY 10457, USA; ^2^Department of Medicine, BronxCare Hospital Center, Bronx, NY 10457, USA; ^3^Department of Pathology, BronxCare Hospital Center, Bronx, NY 10457, USA; ^4^Division of Pulmonary and Critical Care, Department of Medicine, BronxCare Hospital Center, Bronx, NY 10457, USA

## Abstract

**Introduction:**

Human immunodeficiency virus (HIV) positive individuals with the CD4 count less than 200 cells/mm^3^ are at risk for opportunistic infections. *Pneumocystis jirovecii*, a fungal pathogen, is a common cause of opportunistic infections with predominantly pulmonary involvement. Disseminated *P. jiroveciii* infection presenting with hepatosplenic lesion is extremely rare. *Case Summary*. A 31-year-old male with HIV with and acquired immunodeficiency syndrome (AIDS) presented with diarrhea for 3 weeks. He had splenomegaly and inguinal lymphadenopathy on physical examination. Laboratory parameters revealed anemia and hypoalbuminemia, while stool studies for infectious etiology and fecal leucocyte were negative. Computed tomography (CT) of the chest and abdomen depicted consolidation of the lungs and a large splenic mass. He underwent fiberoptic bronchoscopy with transbronchial biopsy which was consistent with *P. jirovecii* pneumonia. He also had a ultrasound-guided core biopsy of the splenic mass which revealed necrotizing granulomas with *Pneumocystis jirovecii* infection on Grocott-Gomori's methenamine silver (GMS) stain and was initiated on treatment for *P. jirovecii* with sulfamethoxazole with trimethoprim.

**Conclusion:**

Malignancy and atypical infection are key differentials in patients presenting with hepatosplenic lesions. HIV positive patients are at increased risk of AIDS-related lymphoma. Tissue diagnosis is often required for further evaluation. Disseminated *P. jirovecii* presenting with splenic mass and liver lesion is extremely rare.

## 1. Introduction

An opportunistic infection is defined as an infection affecting individuals with weakened immune system. Human immunodeficiency virus (HIV) positive patients with CD4 count below 200/mm^3^ are at high risk of the opportunistic infection [1]. Patients with HIV are at increased risk of lymphoma and often present with the extranodal disease with tumor burden [2]. Acquired immune deficiency syndrome (AIDS) related lymphoma is common in HIV-positive patients, especially in those with lower CD4 count [3].

*Pneumocystis jirovecii*, previously known as *Pneumocystis carinii,* is a common opportunistic fungal pathogen mostly affecting immunocompromised individuals [4, 5]. Pulmonary infection is the most common presentation for *P. jirovecii* infection and is often life-threatening [5–7]. Pharmacological prophylaxis is recommended in patients with CD4 count below 200/mm^3^. However, patients with a high HIV viral load in the absence of antimicrobial prophylaxis can present with a disseminated extrapulmonary infection [4, 8]. Extrapulmonary manifestation is exceedingly rare and requires a thorough evaluation to exclude other etiologies such as lymphoma or other malignancies.

We report a case of disseminated pneumocystis in a patient with AIDS presenting with splenic mass and liver nodules along with concurrent *P. jirovecii* pneumonitis. On our review of literature, splenic involvement of pneumocystis is very rare, and only a few cases have been reported so far [4, 9–12].

## 2. Case Summary

A 31-year-old man was evaluated in the emergency room (ER) of our institution for persistent diarrhea of 3 weeks duration. He also reported abdominal pain along with lightheadedness. He described the abdominal pain as vague, diffuse, nonradiating, and mild in intensity without any precipitating or relieving factors. He reported up to 10 episodes of watery diarrhea which did not contain any blood. He also complained of fever and dysuria. About 3 weeks before the current presentation, the patient had presented to the ER with dysuria, abdominal pain, and intermittent diarrhea. At that time he was admitted and treated for a urinary tract infection (UTI) with ciprofloxacin and later discharged home. As per patient, his diarrhea had worsened after taking the antibiotics and had presented with the current symptoms. There was no nausea, vomiting, constipation, fever, and early satiety or appetite changes. He had a prior history of HIV/AIDS, had not been adherent to antiretroviral therapy or PCP prophylaxis. He had a high viral load and low CD4 count.

He was a current smoker and admitted smoking 4 or more cigarettes per day but denied using alcohol except on social occasions or illicit drugs. He did not have a family history of liver, stomach, or colon cancer. His medications included acetaminophen and ciprofloxacin.

On examination, he was found to have a temperature of 39.1°C, blood pressure of 108/52 mmHg, a pulse rate of 99 beats per minute, and a respiratory rate of 16 breaths per minute. The patient was alert, fully oriented, and communicative. The abdomen was scaphoid, nontender, and nondistended with normal bowel sounds. There was no palpable hepatomegaly; however, the spleen was palpable. Shotty cervical and inguinal lymph nodes were palpated. The rest of the physical examination was unremarkable.

**Figure 1:**
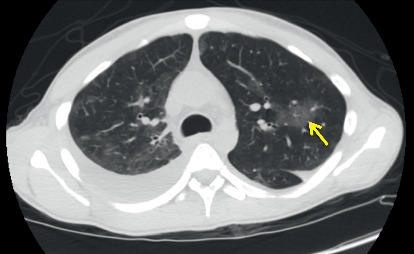
Cross-sectional computerized scan of the chest revealing nodular opacities and ground glass opacities (GGOs) with cavitation in lung field.

**Figure 2:**
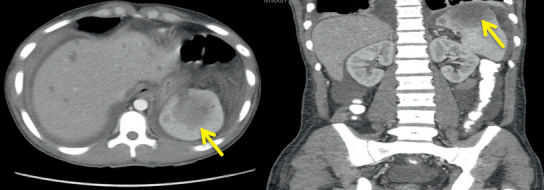
Cross-sectional and coronal computerized tomogram of abdomen with intravenous contrast revealing a hypodense lesion of 6.7 cm in the cephalad aspect of the spleen with adjacent small nodule, multiple liver lesion, and ascites.

**Figure 3:**
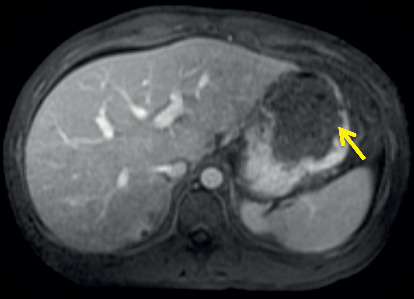
Magnetic resonance imaging of abdomen with gadolinium revealing a heterogeneous lesion in upper pole of the spleen measuring approximately 5.2 × 4.3 cm in size.

**Figure 4:**
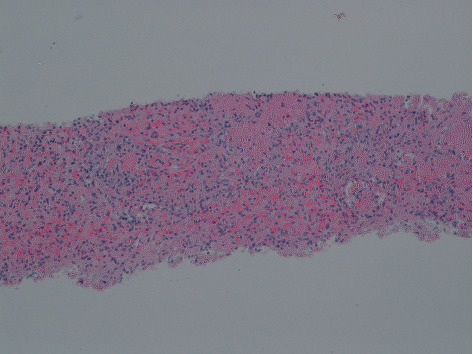
Splenic biopsy with *Pneumocystis jirovecii* infection showing necrotizing granulomatous inflammation/frothy exudate (hematoxylin and eosin stain, magnification ×100 low power).

**Figure 5:**
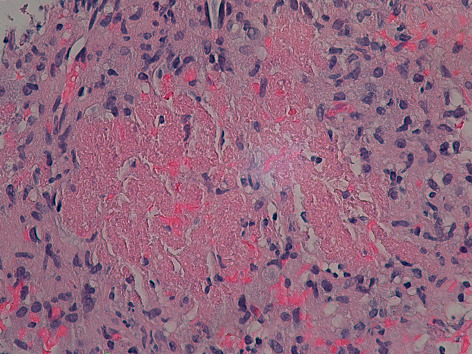
Splenic biopsy with *Pneumocystis jirovecii* infection showing necrotizing granuloma and frothy exudate surrounded by epitheloid cells (hematoxylin and eosin, magnification ×400).

**Figure 6:**
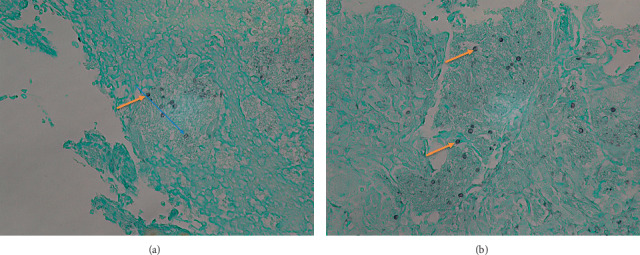
Splenic biopsy on Grocott-Gomori methenamine silver (GMS) stain with 400 times magnification showing the necrotic and frothy infiltrate containing multiple *Pneumocystis jirovecii* organisms (arrow indicates *P. jirovecii*).

Routine laboratory tests showed a hemoglobin level of 8 g/dL, white blood cell count of 3.7 k/uL, serum potassium level of 3.4 mEq/L, and albumin level of 1.7 g/dL. Stool studies including *Clostridium difficile* cytotoxin, fecal leucocyte stain, and fecal fat were negative. His blood and stool cultures were negative for the growth of *Mycobacterium avium* complex. A chest X-ray revealed a right lower lobe consolidation and bilateral pleural effusions. The patient was initiated on broad spectrum antibiotics for hospital-acquired infection. A CT of the chest was done as a part of workup revealed moderate right and small left pleural effusions, consolidation in the dependent lung bases, nodular opacities in both lungs, some cavitary in nature, and patchy ground glass opacity in both lungs (Figure 1). Sputum for acid-fast bacilli (AFB) was negative, ruling out pulmonary tuberculosis. CT abdomen and pelvis done for chronic diarrhea and abdominal pain revealed a hypodense lesion within the cephalad aspect of the spleen, a 6.7 cm splenic mass with adjacent small nodule, multiple hypodense hepatic lesions, measuring up to 12 mm in diameter, and mild ascites in the upper abdomen (Figure 2). Magnetic resonance imaging (MRI) abdomen showed a heterogeneous lesion within the upper pole of the spleen measuring 5.2 × 4.3 cm in size with absent enhancement centrally, with some enhancement along the peripheral aspect of the lesion, suggesting central necrosis (Figure 3). He underwent an ultrasound-guided core biopsy with an 18-gauge (18G) needle. Two core biopsies were obtained for histologic analysis of the splenic mass. The histopathology revealed necrotizing granulomas on hematoxylin and eosin stain (Figures 4 and 5) with *Pneumocystis jirovecii* infection on Grocott-Gomori's methenamine silver (GMS) stain (Figures 6(a) and 6(b)). A fiberoptic bronchoscopy with transbronchial biopsy was also done revealing *P. jirovecii* pneumonia, while bronchoalveolar lavage was negative for malignant cells. His alpha fetoprotein (AFP) level was 3.2 ng/ml. Results of the rest of the laboratory parameters are given in Table 1. The patient was started on sulfamethoxazole with trimethoprim, and no steroids were given. Antiretroviral therapy was not started at the time of discharge due to concerns over IRS (immune reconstitution syndrome). He continued to follow-up at our hospital as an outpatient.

## 3. Discussion

There are a wide array of differentials for a patient presenting with splenic lesion [13]. Angiosarcoma, lymphoma, hemangioendothelioma, and metastasis are the common malignant etiologies of the splenic lesion. MRI of the splenic lesion with and without gadolinium enhancement can assist in further characterization, but is not diagnostic [14]. Image-guided percutaneous biopsy of the spleen with 18G needle is safe and is a favorable alternative to diagnostic splenectomy [15]. It is essential to characterize the vascular pattern of splenic lesion prior to biopsy. Given the risk of malignancy, our patient required splenic biopsy.

Before AIDS pandemic, there were sporadic cases of *P. jirovecii* pneumonia in individuals with immunosuppression due to corticosteroids or chemotherapy [4, 16]. Now, it is the most common respiratory opportunistic infection in AIDS patients. Pneumonia or interstitial pneumonitis is the most common presentation of *P. jirovecii* in HIV/AIDS patients [12]. The first case of fatal extrapulmonary pneumocystis was reported in 1960 [17]. Extrapulmonary or disseminated pneumocystis infection is uncommon, about 0.5% to 2.5% in patients with AIDS [18, 19] but has been described and well documented [8, 20–27].

Disseminated pneumocystis has a highly variable pattern of tissue involvement. It can involve the spleen, liver, gastrointestinal tract [22], skin [28], heart, bone marrow with lymph nodes [8], kidneys, thyroid, and skeletal muscle [24]. The clinical presentation has a wide array of symptoms. Diffuse organ involvement can result in systemic symptoms, whereas local involvement can produce local symptoms, such as isolated involvement of the small intestine [29] causing acute abdomen and thyroid disease, causing left-sided neck swelling and dysphagia [30]. Pneumocystis of the liver has been reported to be associated with hypoalbuminemia, ascites, and anasarca [9, 31, 32].

Splenic involvement of pneumocystis can present with left upper quadrant pain, splenic mass, thrombocytopenia, and splenomegaly [33]. Typical features of splenic pneumocystis on CT abdomen are multiple hypodense lesions with punctate or rim-like calcifications [9, 34].

Our patient with AIDS had presented with abdominal pain, and imaging revealed hypodense lesions in both spleen and liver. Splenic biopsy and transbronchial biopsy revealed pneumocystis consistent with disseminated pneumocystis. Disseminated *P. jirovecii* was diagnosed with splenic and transbronchial lung biopsy, and hence, hepatic biopsy was not required. Although the liver lesions were not biopsied but the clinical presentation of our patient including ascites, hypoalbuminemia and hypodense liver lesions on CT is similar to prior cases reporting hepatic pneumocystis.

## 4. Conclusion

Patients with HIV and AIDS presenting with hepatosplenic mass have a wide array of differentials with opportunistic infection and the malignancy being the most common etiology. Image-guided splenic tissue aspiration is required for the diagnosis. As presented in the index case, disseminated *P. jirovecii* infection can be a possible etiology for the splenic mass.

## Figures and Tables

**Table 1:** Initial laboratory work up.

Parameter	Initial laboratory results	Reference range
Hemoglobin (g/dL)	8.7	12–16
Hematocrit (%)	26.6	42–51
White blood cell count (per mm^3^)	3.7	4.8–10.8
Platelet count (k/*μ*L)	344	150–400
Sodium (mEq/L)	135	135–145
Potassium (mEq/L)	3.4	3.5–5.0
HCO_3_ (mEq/L)	19	24–30
BUN (mg/dl)	6	6–20
Creatinine (mg/dL)	1	0.5–1.5
Glucose (mg/dL)	67	70–120
Lactic acid (mmol/L)	1.5	0.5–1.6
AST (mg/dL)	74	9–48
ALT (mg/dL)	63	5–40
Total bilirubin/direct (mg/dL)	0.2	0.2–1.2
Alkaline phosphatase (U/L)	83	53–141
Albumin (g/dL)	1.8	3.2–4.8
Alpha fetoprotein (AFP) (ng/mL)	3.2	<10
Hepatitis A IgM	Negative	Negative
Hepatitis B core total antibody	Negative	Negative
Hepatitis B surface antibody	Positive	Negative
Hepatitis B surface antigen	Negative	Negative
Hepatitis C antibody	Negative	Negative
HIV RNA PCR (copies/mL)	206,000	Not detectable
Absolute CD4 cells	<20	490–1740
